# Beneficial influence of low-density lipoprotein cholesterol on the endothelium in relation to endothelial repair

**DOI:** 10.1265/ehpm.24-00332

**Published:** 2025-04-05

**Authors:** Yuji Shimizu, Shin-Ya Kawashiri, Hirotomo Yamanashi, Seiko Nakamichi, Naomi Hayashida, Yasuhiro Nagata, Takahiro Maeda

**Affiliations:** 1 Department of General Medicine, Nagasaki University Graduate School of Biomedical Sciences, Nagasaki, Japan; 2 Epidemiology Section, Division of Public Health, Osaka Institute of Public Health, Osaka, Japan; 3 Department of Community Medicine, Nagasaki University Graduate School of Biomedical Sciences, Nagasaki, Japan; 4 Leading Medical Research Core Unit, Nagasaki University Graduate School of Biomedical Sciences, Nagasaki, Japan; 5 Department of Islands and Community Medicine, Nagasaki University Graduate School of Biomedical Sciences, Nagasaki, Japan; 6 Division of Strategic Collaborative Research, Atomic Bomb Disease Institute, Nagasaki University, Nagasaki, Japan

**Keywords:** Atherosclerosis, CD34-positive cell, CAVI, Endothelial repair, LDL, LDL paradox

## Abstract

**Background:**

Low-density lipoprotein cholesterol (LDLc) is regarded as a risk factor for endothelial dysfunction. However, LDLc stimulates the proliferation of hematopoietic stem cells (CD34-positive cells), which contribute to endothelial repair. Therefore, LDLc may have a beneficial influence on the endothelium of individuals with lower endothelial repair activity.

**Methods:**

This cross-sectional study included 245 men aged 60–69 years. Endothelial repair activity was categorized by the circulating levels of CD34-positive cells based on median values. The status of endothelium was evaluated using the cardio-ankle vascular index (CAVI).

**Results:**

Among individuals with low levels of circulating CD34-positive cells, LDL-c levels were significantly inversely correlated with CAVI and positively correlated with circulating CD34-positive cells. No significant correlations were observed among the participants with high levels of circulating CD34-positive cells. Among low levels of CD34-positive cells, the adjusted standardized parameter (β) and *p* value were −0.24 (*p* = 0.021) for CAVI and 0.41 (*p* < 0.001) for CD34-positive cells, whereas among high levels of CD34-positive cells, the corresponding values were 0.03 (*p* = 0.738) and −0.09 (*p* = 0.355).

**Conclusion:**

LDLc has a beneficial influence on endothelial health among individuals with low endothelial repair activity, possibly by stimulating the proliferation of hematopoietic stem cells.

## Graphical Abstract

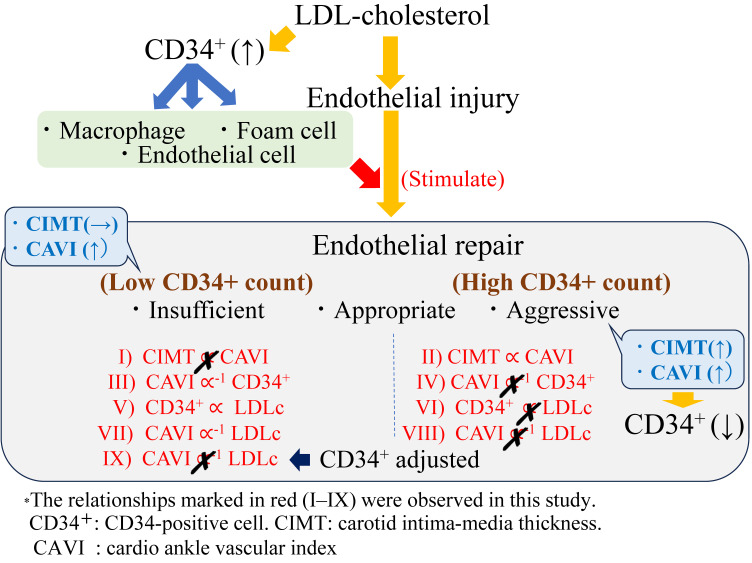

## 1. Introduction

Low-density lipoprotein cholesterol (LDLc) measurement is a common procedure for risk assessment worldwide [1, 2], because high LDLc levels are an established major risk factor for atherosclerotic cardiovascular disease (CVD) [3–5].

However, both high and low LDLc concentrations have recently been reported to be associated with all-cause or CVD mortality [6–8]. The reported association between elevated LDLc and CVD among middle-aged populations differs from that in older populations [9, 10]. Therefore, aging may influence the association between LDLc and all-cause or CVD mortality.

In conjunction with platelets, hematopoietic stem cells, also known as CD34-positive cells, contribute to endothelial repair, partly by differentiating into endothelial cells [11, 12]. Therefore, CD34-positive cells are regarded as endothelial progenitor cells that play a major role in endothelial repair [13]. Endothelial progenitor cells located in vessel walls are crucial for endothelial repair [14].

The number of circulating CD34-positive cells indicates endothelial repair activity [15]. Aging is a process associated with decreasing hematopoietic activity [16], which reduces the number of circulating CD34-positive cells.

Aging is associated with increased oxidative stress [17, 18]. Because oxidative stress induces hypertension [19] and atherosclerosis [20], the need for endothelial repair increases with aging [15]. Therefore, older individuals may have a higher risk of insufficient endothelial repair than younger individuals.

Insufficient endothelial repair, linked to a shortage of CD34-positive cells, progresses to functional atherosclerosis (reduced arterial elasticity), which is evaluated using the cardio-ankle vascular index (CAVI), but not to structural atherosclerosis (increased arterial wall thickness), which is evaluated using the carotid intima-media thickness (CIMT) [21]. Evaluation of the influence of insufficient endothelial repair on the endothelium should be performed using CAVI but not CIMT.

In contrast, aggressive endothelial repair progresses both the functional and structural values of atherosclerosis [21]. Therefore, a positive correlation between the CIMT and CAVI was observed only in individuals with a high capacity for endothelial repair. No correlation was observed between the CIMT and CAVI in patients with a lower capacity for endothelial repair.

In addition, LDLc stimulates the proliferation of CD34-positive cells [22, 23]. Therefore, among those with a lower capacity for endothelial repair and low levels of circulating CD34-positive cells, LDLc could have a beneficial influence on the endothelium by increasing the level of circulating CD34-positive cells.

Based on these correlations, we hypothesized the following: First, significant positive correlation between CIMT and CAVI should only be observed among individuals with high circulating CD34-positive cells. Second, LDLc should be inversely correlated with CAVI only among individuals with low circulating CD34-positive cells. Third, CD34-positive cells should be inversely correlated with CAVI only among individuals with low circulating CD34-positive cells. Finally, LDLc should be significantly correlated with CD34-positive cells only among individuals with low circulating CD34-positive cells.

To evaluate these hypotheses, a cross-sectional study of 245 men aged 60–69 years was conducted.

## 2. Methods

### 2.1 Study population

The methods related to the present risk survey, including CAVI and circulating CD34-positive cell count assessment, have been described previously [15, 21].

This study was approved by the Ethics Committee of Nagasaki University Graduate School of Biomedical Sciences (project registration number: 14051404). Written consent forms were made available in Japanese to ensure a comprehensive understanding of the study objectives. Written informed consent was obtained from all the participants. All procedures involving human participants were performed in accordance with the ethical standards of the Institutional Research Committee, Helsinki Declaration of 1964, and its later amendments for comparable ethical standards.

The study population comprised 319 men aged 60–69 years residing on the Goto Islands in western Japan, who underwent an annual medical checkup between 2013 and 2015, as recommended by the Japanese government. Participants without data on the circulating levels of CD34-positive cells (n = 2) or blood test results (n = 10) were excluded. Participants without CAVI (n = 61) or CIMT (n = 1) data were also excluded. The remaining 245 participants (mean age, 65.4 years; standard deviation [SD], 2.6 years; range, 60–69 years) were enrolled in the study.

### 2.2 Data collection and laboratory measurements

Trained interviewers obtained information on drinking status (non-drinkers, drinks often, or drinks daily) and smoking status (never, former, or current smoker). Body weight and height were measured using an automatic body composition analyzer (BF-220; Tanita, Tokyo, Japan). Body mass index (BMI, kg/m^2^) was calculated. Blood pressure was measured in the right arm after at least 5 min of rest in a sitting position using a blood pressure measuring device (HEM-907; Omron, Kyoto, Japan) and was recorded by trained observers. Blood samples were collected in heparin sodium, siliconized, and sodium fluoride tubes. CD34-positive cells were measured in freshly drawn blood samples from heparin sodium tubes within 24 h of sample collection using BD Trucount™ technology (Becton Dickinson Biosciences, San Jose, CA, USA), an accurate and reproducible single-platform assay cited in the International Society of Hematotherapy and Graft Engineering guidelines [24], and supported by automated software in the BD FACSCanto™ II system.

Approximately 30 min were required to measure the number of CD34-positive cells in each sample. The measurement of CD34-positive cells requires fresh samples within 24 h after blood collection. Since only a limited number of FACSCanto™ II systems were available in our area, a maximum of 20 samples could be processed for CD34-positive cell counts each day. Therefore, we limited the measurement of CD34-positive cells to men aged 60–69 years, who participated in our general health checkups. A detailed description of the circulating CD34-positive cell measurement in our study has been described previously [15, 25].

Serum triglyceride (TG), serum high-density lipoprotein cholesterol (HDLc), LDLc, hemoglobin A1c (HbA1c), and serum creatinine levels were also measured at SRL, Inc., using standard laboratory procedures. The glomerular filtration rate (GFR) was estimated using an established method recently adapted by a working group of the Japanese Chronic Kidney Disease Initiative [26]: GFR (mL/min/1.73 m^2^) = 194 × (serum creatinine [enzyme method])^−1.094^ × (age)^−0.287^.

An experienced vascular examiner measured the left and right carotid arteries. Using a LOGIQ Book XP with a 10-MHz transducer (GE Healthcare), experienced vascular examiners measured the carotid intima-media thickness (CIMT) of the left and right common carotid arteries. The maximum CIMT of each common carotid artery was determined using semi-automated digital edge-detection software (Intimascope; MediaCross, Tokyo, Japan) using a previously described protocol [27]. This software recognizes the edges of the internal and external membranes of the artery semi-automatically and automatically and determines the width at the subpixel level (estimated to be 0.01 mm) [28].

CAVI was determined using a Vasera VS-1000 vascular screening system (Fukuda Denshi, Tokyo, Japan) with the participant in the supine position. The underlying principles of CAVI were described by Yamabe et al. [29].

### 2.3 Statistical analysis

Characteristics of the study population stratified by circulating level of CD34-positive cells (<median, ≥median) were expressed as means ± SD for continuous variables, except for TG. Because TG had skewed distributions, data were expressed as medians [interquartile range], followed by a logarithmic transformation. For daily drinkers, frequent drinkers, current smokers, and former smokers, n (%) was reported. A trend test was performed using a regression model to calculate the *p* value for each variable based on the circulating levels of CD34-positive cells.

To evaluate circulating CD34-positive cell levels, specific correlations between CAVI and other variables, including LDLc, CIMT, and CD34-positive cells, simple correlation analysis, simple linear regression analysis, and multiple linear regression analysis adjusted for relevant confounding factors were performed.

In the multiple regression model, two adjustment models were used to evaluate the correlation between CAVI and LDLc stratified by circulating CD34-positive cell levels. The first model (Model 1) included age, systolic blood pressure (SBP), daily drinker, frequent drinker, current smoker, former smoker, antihypertensive medication use, glucose-lowering medication use, antihyperlipidemic medication use, BMI, HDLc, TG, HbA1c, and GFR. In addition to the variables in Model 1, the next model (Model 3) included CD34-positive cells.

In the multiple regression model, two adjustment models were used to evaluate the correlation between CAVI and CD34-positive cells stratified by circulating CD34-positive cell levels. The first model (Model 2) included age, SBP, daily drinkers, frequent drinkers, current smokers, former smokers, antihypertensive medication use, glucose-lowering medication use, antihyperlipidemic medication use, BMI, HDLc, TG, HbA1c, and GFR. In addition to the variables in Model 2, the next model (Model 3), included LDLc.

All statistical analyses were performed using SAS for Windows (version 9.4; SAS Inc., Cary, NC, USA). Statistical significance was set at *p* < 0.05.

## 3. Results

Among the study population, 122 participants were below the median value of circulating CD34-positive cells (<0.95 cells/µL), whereas 123 participants had more than or equal to the median value of circulating CD34-positive cell (≥0.95 cells/µL).

### 3.1 Characteristics of the study population based on the circulating CD34-positive cell levels

Table 1 shows the specific characteristics of the study population according to the circulating CD34-positive cell levels. Individuals with high levels of CD34-positive cells exhibited significantly higher LDLc levels, a higher prevalence of anti-hypertensive medication use, and lower GFR than those with low levels of CD34-positive cells.

**Table 1:** Characteristics of the study population by CD34-positive cell levels

	**Circulating CD34-positive cells, cells/µL**		**p value**

	**Low** **(<0.95 cells/µL)**	**High** **(≥0.95 cells/µL)**	
No. of participants	122	123	
Age, years	65.6 ± 2.6	65.3 ± 2.7	0.303
Height, cm	164.6 ± 6.3	164.1 ± 5.1	0.493
SBP, mmHg	135 ± 16	138 ± 17	0.181
DBP, mmHg	86 ± 11	87 ± 10	0.382
Daily drinker, %	45.9	49.6	0.565
Frequent drinker, %	15.6	18.7	0.518
Current smoker, %	24.6	28.5	0.495
Former smoker, %	45.9	48.0	0.747
Anti-hypertensive medication, %	40.2	54.5	0.025
Glucose lowering medication, %	9.8	8.9	0.812
Anti-hyperlipidemic medication, %	12.3	13.8	0.724
BMI, kg/m^2^	23.2 ± 3.1	24.0 ± 2.7	0.051
HDLc, mg/dL	58 ± 15	57 ± 14	0.772
LDLc, mg/dL	109 ± 29	120 ± 30	0.006
TG, mg/dL	89 [65, 111]*^1^	95 [67, 137]*^1^	0.059*^2^
HbA1c, %	5.6 ± 0.6	5.8 ± 0.6	0.053
GFR, mL/min/1.73 m^2^	75.9 ± 15.4	71.5 ± 12.7	0.025
CIMT, mm	0.70 ± 0.12	0.69 ± 0.12	0.448
CAVI	8.55 ± 1.00	8.55 ± 0.96	0.997

Values: mean ± standard deviation. *^1^Median values [the first quartile, the third quartile]. Regression model for mean values was used for determining p values. *^2^Logarithmic transformation was used for evaluating p.

BMI, body mass index; CAVI, cardio-ankle vascular index; CIMT, carotid intima-media thickness; DBP, diastolic blood pressure; GFR, glomerular filtration rate; HbA1c, Hemoglobin A1c; HDLc, high-density lipoprotein cholesterol; LDLc, low-density lipoprotein cholesterol; SBP, systolic blood pressure; TG, triglycerides.

### 3.2 Simple correlation between CAVI and targeted variable stratified based on the circulating CD34-positive cell levels

Table 2 presents the results of the simple correlation analysis of the targeted variables. Only in individuals with low circulating CD34-positive cells, LDLc and CD34-positive cells were significantly inversely correlated with CAVI. CIMT was significantly and positively correlated with CAVI, limited to high levels of circulating CD34-positive cells.

**Table 2:** Correlation between CAVI and other variables by circulating CD34-positive cell levels

	**Low ** **(<0.95 cells/µL)**		**High ** **(≥0.95 cells/µL)**	

	**r**	**p**	**r**	**p**
No. of participants	122		123	
Age	0.06	0.510	0.31	0.001
SBP	0.35	<0.001	0.33	<0.001
Daily drinker	−0.02	0.798	0.18	0.0496
Frequent drinker	0.03	0.757	−0.13	0.137
Current smoker	0.07	0.454	0.0004	0.997
Former smoker	0.05	0.570	0.21	0.023
Anti-hypertensive medication	0.08	0.357	−0.05	0.589
Glucose lowering medication	0.05	0.613	0.07	0.472
Anti-hyperlipidemic medication	0.06	0.542	0.02	0.829
BMI	−0.05	0.423	0.04	0.633
HDLc	−0.09	0.336	−0.06	0.540
LDLc	−0.22	0.014	0.01	0.914
TG	0.12	0.204	0.07	0.458
HbA1c	0.19	0.034	0.07	0.458
GFR	0.01	0.941	−0.13	0.149
CIMT	0.07	0.472	0.23	0.010
CD34-positive cell	−0.22	0.014	−0.002	0.984

BMI, body mass index; CAVI, cardio-ankle vascular index; CIMT, carotid intima-media thickness; GFR, glomerular filtration rate; HbA1c, Hemoglobin A1c; HDLc, high-density lipoprotein cholesterol; LDLc, low-density lipoprotein cholesterol; r, simple correlation coefficient; SBP, systolic blood pressure; TG, triglycerides. TG and CD34-positive cells are calculated as logarithmic values.

### 3.3 Circulating CD34-positive cell-level-specific correlations using a simple linear regression model

**Fig. 1:**
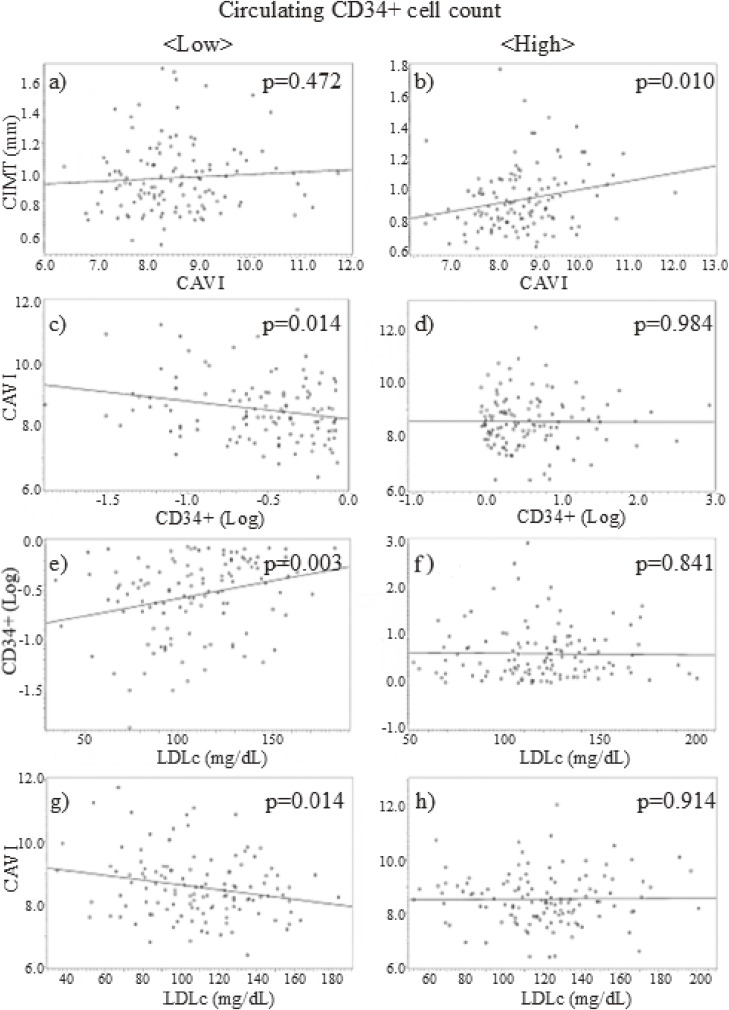
Circulating CD34-positive cell-level-specific correlations using the simple liner regression model Correlations a), c), e), and g) were observed in individuals with low circulating CD34-positive cell level. Correlations b), d), f), and h) were observed in individuals with high circulating CD34-positive cell level. CIMT, carotid intima-media thickness; CAVI, cardio-ankle vascular index; CD34+, CD34-positive cell; Log, logarithmic transformation; LDLc, low-density lipoprotein cholesterol.

Figure 1 shows the circulating CD34-positive cell-level-specific correlations using a simple linear regression model. Only in patients with high levels of circulating CD34-positive cells, CIMT was significantly and positively correlated with CAVI. Moreover, CD34-positive cell was inversely correlated with CAVI only in patients with low levels of circulating CD34-positive cells. In addition, LDL was significantly positively correlated with CD34-positive cell and inversely correlated with CAVI only in patients with low levels of circulating CD34-positive cells.

### 3.4 Multivariable correlations between CAVI and targeted variables among individuals with low level of circulating CD34-positive cells

Table 3 shows the multivariable correlation between CAVI and other variables using the three adjusted models among individuals with low circulating CD34-positive cell levels. Independent of known confounding factors, except for CD34-positive cells (Model 1), LDLc was significantly inversely correlated with CAVI. However, after further adjustment for CD34-positive cells, the statistical power became non-significant (Model 3).

**Table 3:** CAVI and other variables among individuals with low circulating CD34-positive cell level

	**Model 1**		**Model 2**		**Model 3**	
		
	**β**	***p***	**β**	***p***	**β**	***p***
No. of participants	122					
Age	0.05	0.595	−0.0004	0.996	0.03	0.780
SBP	0.31	0.004	0.41	<0.001	0.36	0.001
Daily drinker	−0.05	0.623	−0.04	0.663	−0.07	0.505
Frequent drinker	0.09	0.361	0.09	0.377	0.08	0.426
Current smoker	0.02	0.823	0.02	0.857	0.03	0.780
Former smoker	0.01	0.896	0.01	0.895	0.02	0.852
Anti-hypertensive medication	0.02	0.828	−0.01	0.955	−0.001	0.996
Glucose lowering medication	−0.09	0.397	0.02	0.822	−0.03	0.801
Anti-hyperlipidemic medication	−0.01	0.890	0.04	0.653	0.00	0.998
BMI	−0.20	0.040	−0.17	0.076	−0.17	0.074
HDLc	−0.07	0.500	−0.004	0.970	−0.03	0.765
LDLc	−0.24	0.021	-	-	−0.15	0.163
TG	0.05	0.619	0.05	0.617	0.07	0.538
HbA1c	0.20	0.075	0.10	0.371	0.14	0.222
GFR	−0.01	0.884	0.07	0.442	0.04	0.678
CD34-positive cell	-	-	−0.26	0.005	−0.21	0.033

β, standardized parameter estimate; BMI, body mass index; CAVI, cardio-ankle vascular index; GFR, glomerular filtration rate; HbA1c, Hemoglobin A1c; HDLc, high-density lipoprotein cholesterol; LDLc, low-density lipoprotein cholesterol; SBP, systolic blood pressure; TG, triglycerides. TG and CD34-positive cells are calculated as logarithmic values.

Independent of known confounders, CD34-positive cells were significantly and inversely correlated with CAVI (Model 2). Even after further adjustment for LDLc, the correlation between CD34-positive cells and CAVI remained significant (Model 3).

### 3.5 Multivariable correlations between CAVI and targeted variables among individuals with high level of circulating CD34-positive cells

Table 4 shows the multivariable correlation between CAVI and other variables using the three adjusted models among individuals with high circulating CD34-positive cell levels. No significant correlations were observed between CAVI and LDLc or between CAVI and CD34-positive cells.

**Table 4:** CAVI and other variables among individuals with high circulating CD34-positive cell level

	**Model 1**		**Model 2**		**Model 3**	
		
	**β**	***p***	**β**	***p***	**β**	***p***
No. of participants	123					
Age	0.29	0.001	0.28	0.002	0.29	0.002
SBP	0.34	<0.001	0.34	<0.001	0.34	<.0001
Daily drinker	0.11	0.313	0.11	0.310	0.11	0.305
Frequent drinker	0.01	0.951	0.01	0.961	0.01	0.939
Current smoker	0.25	0.026	0.25	0.026	0.25	0.025
Former smoker	0.24	0.031	0.24	0.030	0.24	0.031
Anti-hypertensive medication	−0.09	0.275	−0.10	0.256	−0.09	0.264
Glucose lowering medication	0.04	0.655	0.04	0.703	0.04	0.686
Anti-hyperlipidemic medication	0.01	0.950	−0.01	0.953	0.00	0.990
BMI	−0.02	0.837	−0.02	0.794	−0.02	0.808
HDLc	−0.05	0.606	−0.05	0.604	−0.05	0.609
LDLc	0.03	0.738	-	-	0.03	0.769
TG	0.06	0.491	0.08	0.424	0.07	0.458
HbA1c	0.05	0.617	0.06	0.558	0.06	0.584
GFR	−0.11	0.228	−0.12	0.201	−0.11	0.208
CD34-positive cell	-	-	−0.04	0.649	−0.04	0.671

β, standardized parameter estimate; BMI, body mass index; CAVI, cardio-ankle vascular index; GFR, glomerular filtration rate; HbA1c, Hemoglobin A1c; HDLc, high-density lipoprotein cholesterol; LDLc, low-density lipoprotein cholesterol; SBP, systolic blood pressure; TG, triglycerides. TG and CD34-positive cells are calculated as logarithmic values.

### 3.6 Multivariable correlations between CD34-positive cells and targeted variables stratified according to circulating CD34-positive cell levels

Table 5 shows the multivariable correlation between CD34-positive cells and other variables, stratified by circulating CD34-positive cell levels. LDLc was significantly positively correlated with CD34-positive cells only in individuals with low levels of circulating CD34-positive cells.

**Table 5:** Correlation between circulating CD34-positive cells and other variables by circulating CD34-positive cell levels

	**Circulating CD34-positive cell level**							

	**Low level (<0.95 cells/µL)**				**High level (≥0.95 cells/µL)**			
	
	**r**	**p**	**β**	***p***	**r**	**p**	**β**	***p***
No. of participants	122				123			
Age	−0.07	0.447	−0.11	0.218	−0.10	0.262	−0.08	0.407
SBP	0.11	0.230	0.27	0.011	−0.002	0.982	0.04	0.663
Daily drinker	−0.06	0.480	−0.08	0.424	−0.002	0.981	0.06	0.591
Frequent drinker	−0.02	0.837	−0.06	0.530	0.03	0.717	0.04	0.704
Current smoker	0.04	0.693	0.03	0.803	0.16	0.084	0.10	0.405
Former smoker	0.01	0.916	0.03	0.795	−0.09	0.343	0.01	0.946
Anti-hypertensive medication	−0.02	0.835	−0.11	0.283	−0.10	0.260	−0.08	0.422
Glucose lowering medication	0.07	0.437	0.30	0.005	−0.03	0.724	−0.11	0.344
Anti-hyperlipidemic medication	−0.10	0.267	0.07	0.498	−0.12	0.202	−0.12	0.230
BMI	0.10	0.295	0.13	0.167	−0.04	0.670	−0.10	0.337
HDLc	0.03	0.729	0.19	0.071	−0.05	0.608	0.01	0.961
LDLc	0.27	0.003	0.41	<0.001	−0.02	0.841	−0.09	0.355
TG	0.05	0.554	0.06	0.599	0.16	0.069	0.17	0.113
HbA1c	−0.05	0.600	−0.29	0.009	0.07	0.448	0.15	0.184
GFR	0.14	0.135	0.26	0.007	−0.17	0.057	−0.19	0.062

BMI, body mass index; CAVI, cardio-ankle vascular index; CIMT, carotid intima-media thickness; GFR, glomerular filtration rate; HbA1c, Hemoglobin A1c; HDLc, high-density lipoprotein cholesterol; LDLc, low-density lipoprotein cholesterol; SBP, systolic blood pressure; TG, triglycerides; r, simple correlation coefficient; β, standardized parameter estimate; p, p for simple correlation coefficient; *p*, p for multivariable linear regression models. TG and CD34-positive cells are calculated as logarithmic values.

For the sensitivity analysis, we re-ran the primary analysis for patients who did not use anti-hyperlipidemic medication, and the same correlations were observed.

In addition, since an individual’s height can influence the productivity of their CD34-positive cells [30] and LDLc levels [31], we also reran the primary analysis adjusted for height level, and the same correlations were observed.

## 4. Discussion

The major findings of this study are that LDLc was significantly inversely correlated with CAVI and positively correlated with CD34-positive cells in individuals with lower endothelial repair activity.

Our previous cross-sectional study with 1,458 older individuals aged 60–79 years revealed an inverse association between LDLc and functional atherosclerosis evaluated as a CAVI of at least 9.0 [32]. This previous study is partly compatible with the present study, which showed a significant inverse correlation between LDLc and CAVI in individuals with lower endothelial repair activity. However, the biological mechanisms underlying the present results have not yet been elucidated.

Hematopoietic stem cell, also known as CD34-positive cells, play a major role in endothelial maintenance [11, 12]. Circulating levels of CD34-positive cells indicate endothelial repair activity when categorized based on median values; individuals with high levels of CD34-positive cells have higher endothelial repair activity than individuals with low levels of CD34-positive cells [21, 30, 33, 34]. Because an inverse correlation between LDL and CAVI was observed only among individuals with low levels of circulating CD34-positive cells, lower endothelial repair activity should be a requirement for LDLc to have a beneficial influence on endothelial health.

**Fig. 2:**
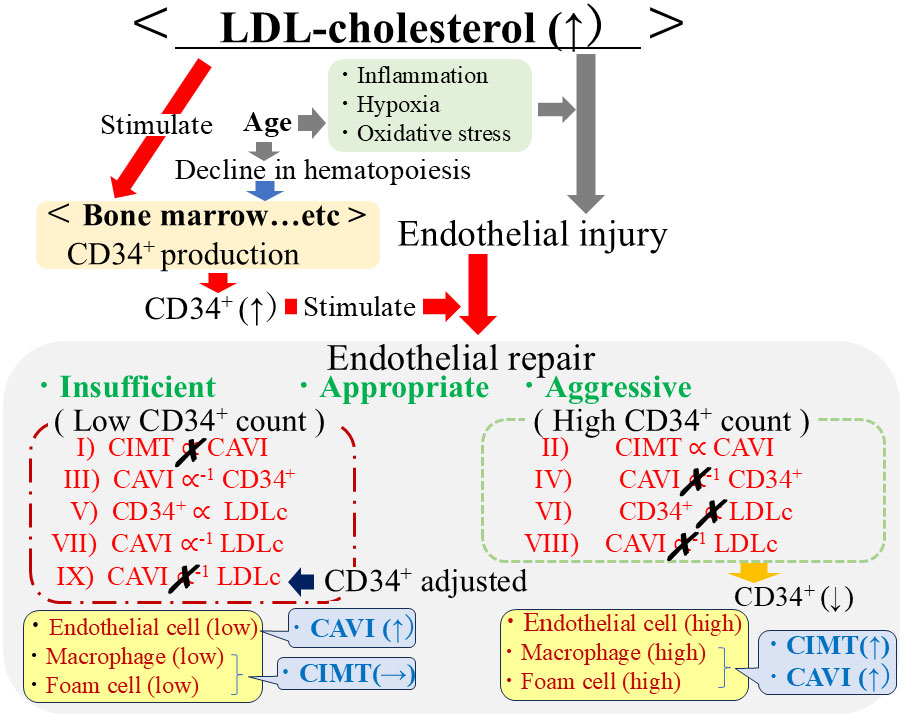
Potential mechanisms underlying the present results The relationships marked in red (I–IX) were observed in this study. CIMT, carotid intima-media thickness; CAVI: cardio-ankle vascular index; CD34+, CD34-positive cell; LDLc, low-density lipoprotein cholesterol.

The potential mechanisms underlying the present results are shown in Fig. 2. The relationships marked in red (I–IX) were observed in this study.

LDLc plays two important roles in the endothelium: endothelial injury, which is related to oxidative stress [35], and endothelial repair, which stimulates the proliferation of CD34-positive cells [22, 23]. Macrophages and foam cells are necessary for the aggressive endothelial repair which relates to progression of CIMT [36, 37]. Aggressive endothelial repair increases both CAVI and CIMT while insufficient endothelial repair related to low circulating CD34-positive cell level increases CAVI but not CIMT [21].

CD34-positive cells differentiate into endothelial cells when endothelial repair is activated [11, 12]; however, they also differentiate into macrophages [38] and foam cells [12, 38]. To reduce oxidative stress in the arterial wall, macrophages phagocytose oxidative LDLc and regulate foam cell formation [35, 39]. Since macrophages and foam cells are known contributors to atherosclerotic lesion development [36, 37], determining the structural value of atherosclerosis, which can be evaluated using CIMT, requires sufficient numbers of circulating CD34-positive cells [40]. Therefore, the analysis of individuals with high circulating CD34-positive cell levels emphasizes the influence of aggressive endothelial repair, whereas the analysis of individuals with low circulating CD34-positive cell levels emphasizes the influence of insufficient endothelial repair [21]. Aggressive endothelial repair increases both the functional and structural value of atherosclerosis, whereas insufficient endothelial repair increases functional atherosclerosis, but not structural atherosclerosis [21, 41]. Since CAVI indicates functional atherosclerosis and CIMT indicates structural atherosclerosis, CAVI showed a significant positive correlation with CIMT only among individuals with high circulating CD34-positive cell levels (Table 2, Fig. 1a and b, Fig. 2I and II).

In addition, during aggressive endothelial repair, the production of CD34-positive cells increases, and many CD34-positive cells become CD34-negative because many of these cells differentiate into endothelial cells, macrophages, and foam cells [11, 12, 38]. This reduction in circulating CD34-positive cells was not observed in patients without aggressive endothelial repair. Furthermore, a shortage of CD34-positive cells increases functional atherosclerosis only in those without aggressive endothelial repair. Therefore, CD34-positive cells showed significantly inverse correlation with CAVI in only individuals with low circulating CD34-positive cell levels (Table 2, 3, 4; Fig. 1c and d; Fig. 2III and IV) [21].

Hypertension is a well-known condition that injures the endothelium and may stimulate the production of CD34-positive cells. However, in the present study, similar values were observed for both low and high levels of circulating CD34-positive cells. Compared with patients with low levels of circulating CD34-positive cells, a higher prevalence of anti-hypertensive medication use was observed among those with high levels of circulating CD34-positive cells. This finding is, in part, consistent with a previous study that reported that anti-hypertensive medication increases CD34-positive cells in normotensive patients with coronary artery disease [42].

Recent studies have shown that LDLc stimulates the production of CD34-positive cells [22, 23]. However, aggressive endothelial repair may mask the effect of LDLc stimulation on CD34-positive cell production, partly because CD34-positive cells often differentiate into mature cells (CD34-negative cells). Therefore, only among individuals with low levels of circulating CD34-positive cells were CD34-positive cells positively correlated with LDLc (Table 5, Fig. 1e and f, Fig. 2V and VI). In individuals with lower productivity of CD34-positive cells, LDLc may act as an indicator of endothelial repair activity, which may have a beneficial influence on CAVI. Subsequently, LDLc was significantly inversely correlated with CAVI in only individuals with low levels of circulating CD34-positive cells (Table 2, 3, 4; Fig. 1g and h; Fig. 2VII and VIII). In addition, possibly because circulating CD34-positive cells mediate the correlation between LDLc and CAVI, after adjusting for CD34-positive cells, the statistical value observed between LDLc and CAVI became non-significant (Table 3, Fig. 2IX).

Although LDLc might have a beneficial influence on endothelial health [32], possibly by stimulating CD34-positive cell proliferation [22, 23], the beneficial influence of high levels of LDLc may be limited because it was only observed among participants with low levels of circulating CD34-positive cells (Table 2, 3, 4; Fig. 1g and h; Fig. 2VII and VIII). LDLc levels were significantly higher among those with high CD34-positive cell level than among those with low CD34-positive cell level (Table 1).

The clinical implication of the present study is that when evaluating the influence of LDLc on endothelial health, the balance between the producibility of CD34-positive cells [22, 23] and endothelial injury [43] should be considered. The CAVI, which is used to evaluate functional atherosclerosis, is a risk marker for cardiovascular diseases [44]. Because a shortage of CD34-positive cells can influence functional atherosclerosis, but not structural atherosclerosis [21], a marker of functional instead of structural atherosclerosis may be appropriate when evaluating the beneficial effects of LDLc on endothelial health.

In the present study, multifaceted analyses were performed that enabled us to observe the potential mechanisms underlying the main results. Therefore, the present study not only shows the circulating CD34-positive cell level-specific correlation between LDLc and CAVI but also shows the potential mechanism underlying these correlations. This is one of the strengths of this study.

The limitations of this study warrant further investigation. First, tissue hypoxia, oxidative stress, and chronic inflammation may play an important role in the correlation between LDLc and endothelial health; however, data on these topics are missing. Further investigations with data on hypoxia-inducible factors, thiobarbituric acid-reactive substances, malondialdehyde, isoprostanes, and high-sensitivity C-reactive protein are necessary. Due to technical and financial problems, no data were available for women. Further investigations in women, with data on circulating CD34-positive cells, are necessary. This study aimed to clarify the biological mechanisms underlying the beneficial influence of LDLc on vascular health among older adults. A previous study showed similar associations between LDLc and functional atherosclerosis in older adults [32]. In addition, a multifaceted analysis can be used to visualize the potential mechanisms underlying these results.

## 5. Conclusion

LDLc has a beneficial influence on endothelial health in individuals with low endothelial repair activity, possibly by stimulating the proliferation of hematopoietic stem cells, known as CD34-positive cells.

## Abbreviations

LDLc: low-density lipoprotein cholesterol
CAVI: cardio-ankle vascular index
β: standardized parameter estimate
CVD: cardiovascular disease
CIMT: carotid intima-media thickness
BMI: body mass index
ISHAGE: International Society of Hematotherapy and Graft Engineering
TG: triglycerides
HDLc: high-density lipoprotein cholesterol
HbA1c: hemoglobin A1c
GFR: glomerular filtration rate
SBP: systolic blood pressure
r: simple correlation coefficient

## Declarations

### Availability of data and materials

For our studies described in this review, we cannot publicly provide individual data due to participant confidentiality, according to ethical guidelines in Japan. Additionally, obtaining informed consent does not include a provision for publicity sharing data. Qualifying researchers may apply to access a minimal dataset. Please contact the office of data management at ritouken@vc.fctv-net.jp. Information for where the data request is also available at http://www.med.nagasaki-u.ac.jp/cm/.
